# Association of Genetic Polymorphisms of Renin–Angiotensin–Aldosterone System-Related Genes with Arterio-Venous Fistula Malfunction in Hemodialysis Patients

**DOI:** 10.3390/ijms17060833

**Published:** 2016-05-27

**Authors:** Yu-Wei Chen, Yu-Te Wu, Jhin-Shyaun Lin, Wu-Chang Yang, Yung-Ho Hsu, Kuo-Hua Lee, Shou-Ming Ou, Yung-Tai Chen, Chia-Jen Shih, Pui-Ching Lee, Chia-Hao Chan, Ming-Yi Chung, Chih-Ching Lin

**Affiliations:** 1 Division of Nephrology, Department of Internal Medicine, Shuang-Ho Hospital, Taipei Medical University, New Taipei 235, Taiwan; b101091063@tmu.edu.tw (Y.-W.C.); yhhsu@tmu.edu.tw (Y.-H.H.); 2 Department of Internal Medicine, School of Medicine, College of Medicine, Taipei Medical University, Taipei 110, Taiwan; 3 Division of Nephrology, Department of Medicine, Taipei Veterans General Hospital, Taipei 112, Taiwan; wyt0406@gmail.com (Y.-T.W.); wcyang@vghtpe.gov.tw (W.-C.Y.); dadabim3520@gmail.com (K.-H.L.); okokyytt@gmail.com (S.-M.O.); box741029@yahoo.com.tw (C.-H.C.); 4 School of Medicine, National Yang-Ming University, Taipei 112, Taiwan; jhshlin0521@gmail.com; 5 Division of Nephrology, Department of Medicine, Taipei City Hospital, He-Ping Branch, Taipei 100, Taiwan; ytchen0117@gmail.com; 6 Division of Nephrology, Department of Medicine, Yuan-Shan Branch, Taipei Veterans General Hospital, I-Lan 264, Taiwan; b001089010@tmu.edu.tw; 7 Department of Medicine, Taipei Veterans General Hospital, Taipei 112, Taiwan; pclee@vghtpe.gov.tw; 8 Institute of Genome Sciences, National Yang-Ming University, Taipei 112, Taiwan; 9 Department of Medical Research, Taipei Veterans General Hospital, Taipei 112, Taiwan

**Keywords:** hemodialysis, arteriovenous fistula, thrombosis, angiotensin receptor gene, single nucleotide polymorphism

## Abstract

Hemodialysis (HD) is the most commonly-used renal replacement therapy for patients with end-stage renal disease worldwide. Arterio-venous fistula (AVF) is the vascular access of choice for HD patients with lowest risk of infection and thrombosis. In addition to environmental factors, genetic factors may also contribute to malfunction of AVF. Previous studies have demonstrated the effect of genotype polymorphisms of angiotensin converting enzyme on vascular access malfunction. We conducted a multicenter, cross-sectional study to evaluate the association between genetic polymorphisms of renin-angiotensin-aldosterone system and AVF malfunction. Totally, 577 patients were enrolled. Their mean age was 60 years old and 53% were male. HD patients with AVF malfunction had longer duration of HD (92.5 ± 68.1 *vs.* 61.2 ± 51.9 months, *p* < 0.001), lower prevalence of hypertension (44.8% *vs.* 55.3%, *p* = 0.025), right-sided (31.8% *vs.* 18.4%, *p* = 0.002) and upper arm AVF (26.6% *vs.* 9.7%, *p* < 0.001), and higher mean dynamic venous pressure (DVP) (147.8 ± 28.3 *vs.* 139.8 ± 30.0, *p* = 0.021). In subgroup analysis of different genders, location of AVF and DVP remained significant clinical risk factors of AVF malfunction in univariate and multivariate binary logistic regression in female HD patients. Among male HD patients, univariate binary logistic regression analysis revealed that right-side AVF and upper arm location are two important clinical risk factors. In addition, two single nucleotide polymorphisms (SNPs), rs275653 (Odds ratio 1.90, *p* = 0.038) and rs1492099 (Odds ratio 2.29, *p* = 0.017) of angiotensin II receptor 1 (*AGTR1*), were associated with increased risk of AVF malfunction. After adjustment for age and other clinical factors, minor allele-containing genotype polymorphisms (AA and CA) of rs1492099 still remained to be a significant risk factor of AVF malfunction (Odds ratio 3.63, *p* = 0.005). In conclusion, we demonstrated that rs1492099, a SNP of *AGTR1* gene, could be a potential genetic risk factor of AVF malfunction in male HD patients.

## 1. Introduction

Patent and reliable vascular access is of vital importance to end stage renal disease (ESRD) patients who receive hemodialysis [1] as their choice of renal replacement therapy (RRT). Arterio-venous fistula (AVF), in comparison with arterio-venous graft (AVG) and tunneled dialysis catheter (TDC), is the best vascular access in terms of higher blood flow rate, lower infection rate, and longer lifespan [2]. The thrombosis and occlusion of AVFs diminish the dialysis adequacy and increase the morbidity and mortality of HD patients [3]. Neo-intimal hyperplasia (NIH) causes the maturation failure of AVF, while the recurrent thrombosis compromises the patency in the long-term. Excessive strength and duration applied on puncture site, insufficient anticoagulation use and infection of vascular access all contribute to the recurrent thrombosis of AVF. Multiple studies had been conducted to define the genetic risk factors of AVF thrombosis [3,4,5]. Coagulation-related genes, hyperhomocysteinemia-related methylene tetrahydrofolate reductase (*MTHFR*) gene, transforming growth factor-b1 (*TGFB1*), matrix metalloproteinases (MMPs), hypoxia inducible factor-1α (*HIF1A*), heme oxygenase-1 (*HO1*) and vascular endothelial growth factor-A (*VEGFA*) and its receptors (*FLT1* for VEGFR-1 and *KDR* for VEGFR-2) have all been studies for their association with AVF stenosis or thrombosis.

The renin-angiotensin-aldosterone system plays an important role in the regulation of blood pressure and homeostasis of body fluid. The impact of genetic polymorphism of ACE on AVF thrombosis has been studied but contradictory results had been found [6,7,8,9,10,11]. Fewer studies focused on the role of angiotensin II receptor 1/2 (AGTR1/2) in the pathogenesis of AVF thrombosis. The aim of this study was to conduct a case-control study to discover whether single nucleotide polymorphism (SNP) of renin-angiotensin-aldosterone system (RAAS) genes (including Angiotensinogen (*AGT*), Angiotensin converting enzyme gene (*ACE*), angiotensin II receptor 1 (*AGTR1*) and 2 (*AGTR2*)) could be genetic risk factors of AVF thrombosis.

## 2. Results

### 2.1. Patient Characteristics

Totally, 577 ESRD patients were enrolled into our study. The demographic and clinical characteristics of study subjects are shown in Table 1. Among them, 154 patients had AVF malfunction and the other 423 patients were included as control group. The mean age of the AVF malfunction patients and control group patients was 60.7 ± 16.1 and 59.8 ± 14.0 years old, respectively (*p* = 0.517). There was no difference in the proportion of gender between the two groups: 55.2% of AVF malfunction patients were male and 52.2% of control group were male (*p* = 0.531). Vintage of HD was significantly longer in AVF malfunction group than in control group (92.5 ± 68.1 *versus* 61.2 ± 51.9 months, *p* < 0.001). No significant difference between patients with and without AVF malfunction in the frequency of smoking was observed (11.7% *vs.* 9.5%, *p* = 0.432). As to the comorbidity, the prevalence of diabetes mellitus, cerebrovascular accident, peripheral arterial disease and coronary artery disease did not differ between patients with AVF malfunction and control group (Table 1). However, the prevalence of hypertension in AVF malfunction group was significantly lower (44.8% *versus* 55.3%, *p* = 0.025). As to the hemodialysis-related parameters, ESRD patients with AVF malfunction had significantly higher average dynamic venous pressure (DVP) than control group (147.8 ± 28.3 *versus* 139.8 ± 30 mmHg, *p* = 0.021). However, there was no significant difference between patients with and without AVF malfunction in pre-HD mean arterial pressure (104.8 ± 17.6 *vs.* 109.7 ± 19.1 mmHg, *p* = 0.109) and post-HD mean arterial pressure (92.8 ± 14.4 *vs.* 96.6 ± 15.1 mmHg, *p* = 0.184). Delivered dialysis dosage was similar between two groups of patients, in terms of Kt/V and urea reduction rate (URR) (Table 1).

SNPs of RAAS-related genes studied in our study are listed in Table 2. All SNPs tested in our study were within Hardy-Weinburg equilibrium.

### 2.2. Univariate Analysis of the Risk Factor of AVF Malfunction

#### 2.2.1. Clinical and Genetic Risk Factor of AVF Malfunction in all Study Subjects

We included each clinical and demographic characteristic and genotype of each SNP for univariate analysis. Patients with two major alleles are grouped as control and patients with one or two minor alleles are grouped as risk group. Logistic regression was performed and the results are expressed as odds ratio with 95% confidence interval and p value is also listed in Table 3. Right-sided AVF (Odds ratio (OR) 2.064, *p* = 0.001), upper arm location of AVF (OR 3.381, *p* < 0.001) and increasing dynamic venous pressure (OR 1.011 for each increment of 1 mmHg, *p* < 0.001) were statistically significant risk factors for AVF malfunction. Hypertension, in contrast, was a significant protective factor of AVF malfunction (OR 0.656, *p* = 0.026). None of the SNPs of *AGT*, *ACE*, *AGTR1* and *AGTR2* genes were identified as significant risk factor of AVF malfunction. Estrogen has been widely known to possess protective effects on cardiovascular system [12], thus we divided the study subjects according to different genders for further analysis of possible clinical impact of SNPs.

#### 2.2.2. Clinical and Genetic Risk Factor of AVF Malfunction in Female Study Subjects

Upper arm location of AVF (Odds ratio (OR) 2.690, *p* = 0.004) and increasing dynamic venous pressure (OR 1.017 for each increment of 1 mmHg, *p* = 0.001) were the only two risk factors for AVF malfunction in female HD patients (Table 4). Laterality of AVF was not a significant factor for AVF malfunction in female HD patients (OR 0.766, *p* = 0.411). Hypertension seemed to have protective effects on AVF, but there is no statistical significance (OR 0.606, *p* = 0.077). None of the SNPs of *AGT*, *ACE*, *AGTR1* and *AGTR2* genes is identified as significant risk factor of AVF malfunction in female HD patients.

#### 2.2.3. Clinical and Genetic Risk Factor of AVF Malfunction in Male Study Subjects

Among male HD patients in current study, right-sided AVF (OR 3.051, *p* < 0.001) and upper arm location of AVF (OR 4.474, *p* < 0.001) were two risk factors for AVF malfunction. Protective effect of HTN was not observed in male subgroup. Increasing dynamic venous pressure seemed to increase the risk of AVF malfunction, with borderline statistical significance (OR 1.008 for each increment of 1 mmHg, *p* = 0.061). Two SNPs in *AGTR1* gene probably had adverse impacts on AVF patency. The first one is rs275653 (Promotor (g. −113), −153A>G) and male HD patients with minor allele (AG or GG) had higher risk of AVF malfunction than those with major alleles only (GG genotype) (OR 1.900, *p* = 0.038). Another potential genetic risk factor of AVF malfunction is rs1492099 (Intron (IVS2 + 11689)). Male HD patients having minor allele (CA or AA genotype) were associated with higher prevalence of AVF malfunction than those carrying two major alleles (CC genotype) (Table 5). None of SNPs in *AGT* gene, *ACE* gene or *AGTR2* gene was significantly associated with AVF malfunction. However, AG or GG genotype of rs6687360 (Intron(IVS2+749)) of *AGT* gene and TC or CC genotype of rs5182 (Synonymous coding(Exon3, c. +573)) had potential effects on AVF patency; thus, these two SNPs were also included in the following multivariate analysis (OR 1.615, *p* = 0.076 and OR 1.623, *p* = 0.084, respectively, Table 5).

### 2.3. Multivariate Analysis of Risk Factors of AVF Malfunction in Male HD Patients

To define the risk factor of AVF malfunction in male HD patients, we included clinical and genetic parameters with stronger association with AVF malfunction, *i.e.*, *p* < 0.200 in univarate binary logistic regression, and perform multivariate Cox-regression analysis. Forward likelihood ratio test was applied to construct the model. Age, hypertension, laterality of AVF, upper arm/forearm location of AVF, dynamic venous pressure, rs275653, rs1492099, rs6687360, rs5182 and rs409742 are included into analysis. As shown in Table 6, right-sided AVF (OR 3.611, *p* = 0.001), upper arm location of AVF (OR 3.384, *p* = 0.003) and CA or AA genotype of rs1492099 (OR 3.355, *p* = 0.008) remain significantly associated with AVF malfunction after adjustment.

## 3. Discussion

### 3.1. AVF Malfunction, HD Characteristics and Comorbidities

The longer duration of hemodialysis represents much more total needle puncture times. Repeated injury to AVF may induce local inflammation, thrombosis and fibrotic change or stenosis. In our study, hemodialysis patients with AVF malfunction have significantly longer HD duration than control group. The finding is compatible with aforementioned pathophysiology of development of AVF malfunction. In our study population, most of the patients, like other cultural groups in the world, are right-handed.

Cardiovascular surgeons tend to establish AVF anastomosis on the forearm of non-dominant upper extremity, *i.e.*, left forearm. HD patients who had their AVF on dominant side and upper arm might have worse vascular condition [13].

In our study, HD patients without AVF malfunction have significantly higher prevalence of HTN (Table 1). Hypertension is also found to be a protective factor for AVF malfunction in univariate analysis of all study subjects (Table 3). Hemodialysis patients with lower blood pressure may be prone to develop intradialytic hypotension, lower blood flow rate and thus may result in clotting within dialyzer or vascular access. Therefore, HD patients who have HTN may probably benefit from a higher blood pressure, whether during or between hemodialysis sessions. However, protective effects of HTN are not significant among male HD patients. One possible explanation is that the male patients tended to exercise their upper limbs more often, whether in their labor work or recreational activities. Larger muscle girdle may potentially interfere with the measurements of blood pressure.

Compared with HD patients without AVF malfunction, HD patients with AVF malfunction have higher average dynamic venous pressure (Table 1). Higher dynamic venous pressure implicates hindrance of venous return, such as thrombosis or stenosis. Thrombosis and stenosis are both the major mechanical causes of AVF malfunction.

### 3.2. Genetic Risk Factors of AVF Malfunction

Various studies have been conducted to delineate the association between genetic polymorphisms and arteriovenous fistula patency. In our previous study, length polymorphism of dinucleotide guanosine thymine repeat (GT)*n* in the promoter region of heme oxygenase-1 (*HO1*) was found to be associated with AVF patency. Longer GT repeat in promoter region of *HO1* may hinder the transcription of the gene, and eventually attenuate the protective effect of heme oxygenase-1 on AVF [5]. Recent publication from our group also discovered that SNP of protein arginine methyltransferase 1 (*PRMT1*) gene has statistically significant association with primary patency, assisted primary patency and secondary patency of AVF in male HD patients [14].

Clinical implication of genetic polymorphism of *ACE* gene, especially *ACE* insertion/deletion (*ACE* I/D), has been extensively studied [6,7,8,9,10,11]. On microvascular scale, Hadjadj *et al.* [9] conducted a large, family-based, case-control study in 3 different European populations and they discovered that five *ACE* polymorphisms (rs1800764-C, rs4311-T, Insertion/deletion (I/D or rs1799752)-D, rs4366-G, and rs12449782-G alleles) had statistically significant association with increased risk of diabetic nephropathy. However, contradictory results were shown by different studies and ethnic group and gender may contribute to the disparities of results. In a Taiwanese study completed by Tien *et al.* [15], female diabetic patients with *ACE* ID or DD genotype were associated with higher risk of diabetic nephropathy than those with *ACE* II genotype. On macrovascular scale, *ACE* polymorphisms have been found to be associated with cerebrovascular accident [16]. However, a recent Spanish case-control study by Domingues-Montanari *et al.* demonstrated that there was no significant association between *ACE* polymorphism and ischemic stroke, despite elevated serum level of ACE in *ACE* DD genotype group [11]. In our study, none of the selected SNPs of *ACE* gene was associated with AVF malfunction, which was compatible with previous studies [3,6]. In a longitudinal study conducted by Heine *et al.*, 137 HD patients with AVF were enrolled and AVF patency 12 months after creation of AVF were not significantly different among patients with different genotypes (*ACE* II, ID and DD). Effects of angiotensin converting enzyme inhibitor (ACE-I) and angiotensin receptor blocker (ARB) were evaluated and the results showed ACE-I/ARB had no positive effects on AVF patency [6]. However, a Korean study with similar scale but longer follow-up period had the opposite discoveries [17]. Recently, another smaller study revealed possible association between *ACE* DD genotype and higher risk of early thrombosis of AVF in HD patients, in comparison with HD patients with *ACE* II or ID genotype [7].

Genetic polymorphisms of *AGTR1* have been found to be closely related to cardiovascular diseases, metabolic disorders and even longevity in previous studies [18,19,20,21,22]. The most widely studied SNP of *AGTR1* gene, rs5182 (A1166C) is located on the 3′-UTR of AGTR1 gene exon 3. Recently, a meta-analysis by Liu *et al.* included 56 studies and 28,952 study subjects and the study found that *AGTR1* A1166C was associated with higher risk of hypertension, whether in Asian or Caucasian populations [23]. Significant association between *AGTR1* rs5182 and hypertension was also found in Mexican population [19]. Interestingly, previous epidemiology-genomic studies have found the patients with *AGTR1* CC genotype were at increased risk of in stent restenosis (ISR) after percutaneous coronary intervention, whether in Caucasian or Asian ethnic groups [24,25]. However, in our study, there was no statistically significant association between rs5182 and AVF malfunction (Table 3, Table 4 and Table 5). Even it is included into multivariate analysis of male HD patients, it did not appear to be a significant risk factor of AVF malfunction (Table 6). In our study, another 2 SNPs of *AGTR1* gene were found to be associated with AVF malfunction: rs275653 and rs1492099. *AGTR1* rs275653 was included by the gene association study in Mexican population, it turned out that rs275653 was not associated with hypertension [19]. In a gene association study involving Italian centenarian cohort and Japan validation centenarian cohort, rs275653 C-G-C genotype (minor allele) has been found to be associated with longevity, less AGTR1-expressing peripheral blood neutrophils and lower blood pressure [20]. In our study, patients with minor allele of rs275653 were at increased risk of AVF malfunction, probably due to lower blood pressure. *AGTR1* rs1492099 was the other significant risk factor for AVF malfunction. Unfortunately, limited literature revealed the clinical implication of this SNP. Sotoodehnia *et al.* have conducted a case-control study on adverse cardiovascular effects of SNPs of angiotensin-converting enzyme-related genes [18]. In this study, minor allele of rs1492099 had significant association with decreased risk of sudden cardiac death (OR 0.62, 95% CI 0.4–0.9). In our study, male HD patient with rs1492099 minor allele were at increased risk of AVF malfunction (Table 5 and Table 6). The proportion of AVF malfunction in all male HD patients was 26.7%; however, the proportion of AVF malfunction in male HD patients with minor allele of *AGTR1* rs1492099 was 43.9%. Although *AGTR1* rs1492099 locates on intron of *AGTR1* gene, it could possess regulatory effects on transcription of *AGTR1*, which may cause increased/decreased expression. Altered expression of *AGTR1* may cause decreased systemic expression, which may result in lower prevalence of hypertension. In contrast, minor allele of rs1492099 may result in increased expression of *AGTR1*, which renders HD patients become more susceptible to angiotensin II, augmented vascular smooth muscle cell proliferation, neointimal hyperplasia and eventually stenosis and malfunction of AVF [26,27,28]. ACE-I or ARB may be the “double-blade sword.” One possible therapeutic approach is that ACE-I/ARB should be considered as the “first” antihypertensive in HD patients with hypertension, to gain their beneficial effects on inhibition of vascular smooth muscle cell proliferation, meanwhile, to avoid hypotension episodes that may increase the risk of AVF malfunction or thrombosis.

Adverse effects of *AGTR1* SNPs were observed only in male HD patients but not in female HD patients. An animal study of estrogen effects on adrenal gland secretion showed that estrogen repressed the expression of AGTR1 and obtunded the aldosterone release stimulated by angiotensin II [29]. Generally speaking, estrogen may lower the AT1 receptor density and increased AT2 receptor density [30]. Therefore, it is possible that the up-regulation effect of SNPs on AGTR1 are attenuated or “overwritten” by hormone effects in female patients.

### 3.3. Limitations

There are several limitations in our study: First, lack of time dependent variable, such as the prospective follow-up period, makes analysis of causal relationship between clinical/genetic factors and AVF malfunction inapplicable. HD vintage in patients without AVF malfunction is significantly shorter. The actual prevalence of AVF malfunction among control patients when their HD vintage reaches to similar duration as AVF malfunction group is unknown. Second, robust statistical analysis cannot be performed due to relatively small numbers of study subjects. Third, all the study subjects are Taiwanese and the results in our study may not be extrapolated to other ethnic groups. A similar cohort with detailed SNP data in Taiwan may be used to validate our findings, unfortunately, there was no such independent cohort in previously published studies. Fourth, although we advocated the estrogen effect as an interpretation of gender differences on effects of SNPs, further subgroup analysis of premenopausal and menopausal female patients was not available because of limited patient number in our study. Fifth, selection bias may exist in the cross-sectional study design *per se*. Sixth, detailed information on medication use of ACE-I or ARB is not available, thus it is difficult to delineate the true clinical effects of angiotensin or angiotensin receptor. Last, but not least important, no blood chemistry data were available to calculate Kt/V or urea reduction rate and no CRP (C-reactive protein) or albumin level data were available to determine the status of inflammation or malnutrition, respectively.

## 4. Materials and Methods

### 4.1. Study Subjects

All ESRD patients receiving regular HD therapy at eight different medical institutions during January to March 2008 were screened for eligibility of current study. The eight medical institutions included five local hemodialysis centers, two regional hospitals and one medical center. The inclusion criteria were listed as follows: adult age elder than 18 years old, prevalent ESRD patients who received stable HD (HD therapy for more than 3 months), using native AVF at upper extremities. Definition of AVF malfunction in current study is the loss of functional patency (patent AVF which can tolerate extracorporeal blood flow more than 200 mL/min during dialysis), any angioplasty or surgery to re-establish AVF patency and total occlusion of the AVF. To investigate specifically on AVF thrombosis, patients with AVF malfunction which were attributed to complications other than thrombosis are excluded, *i.e.*, patients with vascular access infection, aneurysm formation with or without surgical ligation, and dialysis access-associated steal syndrome. Patients with AVF maturation failure (AVF malfunction within 3 months after surgery) were also excluded. Informed consent was obtained from each participant of the study; clinical information and blood sample were then collected. We recorded demographic and clinical information of all the patients recruited, including age, gender, HD vintage, location (upper arm or forearm) and laterality (right or left) of the AVF. Underlying comorbidities such as hypertension (HTN), diabetes mellitus [31], peripheral arterial occlusive disease (PAOD), cerebrovascular disease (CVA), coronary artery disease (CAD) were all recorded. Hemodialysis-related parameters including maximal blood flow rate during HD and dynamic venous return pressure (DVP) were also documented. The conduction of the study was under the surveillance of the Institutional Research Board of Taipei Veterans General Hospital.

### 4.2. DNA Isolation and Genotyping of Renin-Angiotensin-Aldosterone System-Related Genes

Venous blood samples with anticoagulant were collected from all study subjects and isolation of genomic DNA was done by using Puregene DNA purification kit (Gentra, Minneapolis, MN, USA). Fifty to 100 ng of DNA was amplified into a final volume of 10 μL, which contained 0.5 U HotStar Taqpolymerase (Qiagen, Hilden, Germany), 1× solution Q (Qiagen), 10× reaction buffer (Qiagen), MgCl_2_ (Qiagen), 200 μmol each deoxynucleoside triphosphate (Roche Applied Science, Mannheim, Germany) and 10 μmol of each primer. The protocol of thermal cycler for DNA amplification was the following: 95 °C for 12 min, 30 cycles of 95 °C for 1 min, 60 °C for 30 s, 72 °C for 1 min, 72 °C for 8 min. Electrophoresis with 2% agarose gel was used for fractionation of amplified DNA products. The DNA bands in each lane were visualized by ultraviolet light and ethidium bromide staining (0.5 mg/mL). Table 1 listed the primer sequences of selected SNP in current study. Direct sequencing (allelic discrimination with TaqMan MGB probe by ABI 7700) and restriction fragment length polymorphism (RFLP) were applied to determine the SNP genotyping. Positive and negative controls were included in all typing analysis.

### 4.3. Statistical Analysis

We checked Hardy–Weinberg equilibrium for the genotype polymorphisms [32]. Continuous variables were expressed as mean ± SD and Student’s *t*-test was used to compare differences between groups. Categorical variables were depicted as median with interquartile range and we applied χ-square test to determine differences between groups. Logistic regression was used to define whether each clinical factor or SNP could be the risk factor for AVF malfunction. Multivariate logistic regression model was used for adjustment of confounders and determination of independent risk factors of AVF thrombosis. We performed two-sided tests in statistical analysis and *p* value less than 0.05 was considered to imply statistical significance. Statistical Package for the Social Sciences (SPSS) version 18.0 (SPSS, Chicago, IL, USA) was applied in statistical analysis.

## 5. Conclusions

In the current study, rs1492099, a SNP of *AGTR1* gene, is an independent risk factor for AVF malfunction, even after adjustment of other candidate SNPs and clinical factors. Further well-designed study with larger scale or related animal study is needed to determine the exact effects of this SNP.

## Figures and Tables

**Table 1:** Clinical characteristics of Hemodialysis (HD) patients by status of Arterio-venous fistula (AVF) malfunction.

Characteristic	Malfunction (*n* = 154)	No Malfunction (*n* = 423)	*p* Value
Age (years)	60.7 ± 16.1	59.8 ± 14.0	0.517
Gender (%)			
Male	55.2	52.2	0.531
Female	44.8	47.8	
HD duration (months)	92.5 ± 68.1	61.2 ± 51.9	<0.001
Smoking (%)	11.7	9.5%	0.432
Hypertension (%)	44.8	55.3	0.025
Diabetes mellitus (%)	26.1	31.9	0.173
Cerebrovascular accidents (%)	11.0	7.3	0.192
Peripheral arterial disease (%)	5.2	4.0	0.540
Coronary artery disease (%)	23.0	20.2	0.462
ACE inhibitor (%)	9.7	11.1	0.643
ARB (angiotensin II receptor blocker) (%)	14.9	18.0	0.391
Site of AVF (%)			
Right side	31.8	18.4	0.002
Left side	68.2	81.6	
Location of AVF (%)			
Forearm	73.4	90.3	<0.001
Upper arm	26.6	9.7	
Dynamic venous pressure (mmHg) under pump flow at 250 mL/min	147.8 ± 28.3	139.8 ± 30.0	0.021
Pre-dialytic mean arterial blood pressure (mmHg)	104.8 ± 17.6	109.7 ± 19.1	0.109
Post-dialytic mean arterial blood pressure (mmHg)	92.8 ± 14.4	96.6 ± 15.1	0.184
KT/V	1.38 ± 0.13	1.49 ± 0.15	0.113
URR (urea reduction rate) (%)	73 ± 4.9	76 ± 5.2	0.124
Maximal pump flow (mL/min)	268.6 ± 29.3	274.5 ± 36.3	0.106

HD, hemodialysis; AVF, arteriovenous fistula.

**Table 2:** Primer sequences and PCR conditions for amplification of polymorphisms within renin-angiotensin-aldosterone system-related genes.

Gene	SNP Name Position	Chromosome Position	Genotyping Method	Primer	Sequence (5′ to 3′)	Allele *
*AGT*	rs7079 exon5 (c. +1866)	chr1:230838331	TaqMan Allelic discrimination	Forward	TGAAAGATGCAAGCACCTGAA	C/A *
				Reverse	TTGGAGGCTTATTGTGGCAAG	
	rs11568056 Intron (IVS2-551)	chr1:230842497	RFLP-PCR	Forward	GACAGCTGGTGGGCTCTG	G/A *
				Reverse	(6Fam)-CAAAGGCTGTGGTTTGACAC	
	rs6687360 Intron (IVS2+749)	chr1:230844992	RFLP-PCR	Forward	(6Fam)-GTGCCATCATTGCTCACTGT	T/C *
				Reverse	GGCCTATACAGCCCTCCTCT	
	rs4762 Non-synonymous 207 T>M Exon2, c. +620	chr1:230845977	RFLP-PCR	Forward	(6Fam)-CTACAGGCAATCCTGGGTGT	C/T *
				Reverse	AGGCCTGACTGGCTGATCT	
	rs11568028 Intron (IVS1-645)	chr1:230847244	RFLP-PCR	Forward	(6Fam)-GGACCACAGGGAGATGACAA	G/A *
				Reverse	ATGAGGCCATGAGGGTGA	
	rs3789678 Intron (IVS1+350)	chr1:230849482	RFLP-PCR	Forward	GGACAAGATGGTCAGGTCTTC	C/T *
				Reverse	(6Fam)-TCCCAAAGCTTAGAAAGCACT	
	rs5051 5′-UTR (Exon 1, g. +172, c. −41) -6A>G	chr1:230849872	RFLP-PCR	Forward	GTCCTTCTGGCCAGCCTGT	T/C *
				Reverse	(6Fam)-CGGCCTTTTCCTCCTAGC	
*ACE*	rs4295 Intron (IVS2-70)	chr17:61556298	RFLP-PCR	Forward	(6Fam)-CTGTCCCCCACTCCACAG	C/G *
				Reverse	GACCCTACACAACTGCATGG	
	rs4340 Intron (Intron 16)	chr17:61565893	Fragment length analysis	Forward	(6Fam)-GTAAGCCACTGCTGGAGAGC	I/D *
				Reverse	CCAGCCCTTAGCTCACCTCT	
	rs10853044 3′-UTR (Exon17, c. +5150)	chr17:61586549	RFLP-PCR	Forward	TGGCTAAAGGGTGAGATGGTG	T/C *
				Reverse	(6Fam)-CAGGGACAGACAGGCCAAG	
*AGTR1*	rs409742 Upstream (g. −3294)	chr3:148412365	RFLP-PCR	Forward	TTCCCACCAACAATATATGAGG	T/C *
				Reverse	(6Fam)-AAACGTAGGAGTAAACCTTTGTTACC	
	rs275653 Promotor (g. −113) −153A>G	chr3:148415545	TaqMan Allelic discrimination	Forward	TGAACGCTGATCTGATAGTTGACA	A/G *
				Reverse	ACGAGGCTCTGTTTTGCATTC	
	rs10935724 Intron (IVS1-4477)	chr3:148421253	RFLP-PCR	Forward	CATTTTAGCAAAATCCTCAGGTG	A/C *
				Reverse	(6Fam)-CAGCTTTTGGGTAAACTACTTATCTC	
	rs1492099 Intron (IVS2+11689)	chr3:148437503	RFLP-PCR	Forward	(6Fam)-GCCTGTGCTGTTCTCAGGTT	G/A *
				Reverse	AACTTTAAATGTTTTACAGATCCAAAT	
	rs385338 Intron (IVS2-9620)	chr3:148449156	RFLP-PCR	Forward	(6Fam)-TTTCTTTTGGACAGCACTGAA	C/G *
				Reverse	AATGCAAGGGTAAGTAAAATGAA	
	rs5182 Synonymous coding (Exon3, c. +573)	chr3:148459395	RFLP-PCR	Forward	(6Fam)-CATCATCATTTGGCTGCTG	T/C *
				Reverse	CGTGTCCACAATATCTGCAA	
	rs5186 3′-UTR (Exon3, c. 1166) 1166A>C	chr3:148459988	RFLP-PCR	Forward	GAGAACATTCCTCTGCAGCAC	A/C *
				Reverse	(6Fam)-GAGCAGCCGTCATCTGTCTA	
*AGTR2*	rs1403543 Intron (IVS1-29, g. 218) 1675A>G	chrX:115302192	TaqMan Allelic discrimination	Forward	GCAGCCTGAATTTTGAAGGT	A/G *
				Reverse	TCCACTTGAAGACTTACTGGTTGT	
	rs11091046 3′-UTR (Exon 3, c. +1593) 3123A>C	chrX:115305126	RFLP-PCR	Forward	(6Fam)-CATTGCATCATTTACAAGACAACA	C/A *
				Reverse	ACTGTAAAAATAAGCTAAAGCATAGGA	
	rs12840631 Downstream (g. +4317)	chrX:115306351	TaqMan Allelic discrimination	Forward	TGTATCCTCCATTTTATCTCCACTGA	C/G *
				Reverse	AGCTAATGGGAAATTATGGCTCAA	

* Minor allele, also as risk allele in statistical analysis.

**Table 3:** Univariate logistic regression model of factors associated with AVF malfunction in all HD patients.

Clinical or Genetic Factors	Odds Ratio	95% CI Lower	95% CI Upper	Significance
Age (year)	1.005	0.992	1.018	0.488
Right side *vs.* left side	2.064	1.358	3.138	0.001 *
Upper arm *vs.* forearm	3.381	2.090	5.469	<0.001 *
Hypertension	0.656	0.452	0.950	0.026 *
Diabetes mellitus	0.755	0.499	1.143	0.184
Coronary artery disease	1.183	0.757	1.848	0.462
Peripheral artery disease	1.309	0.553	3.097	0.541
Cerebrovascular accident	1.569	0.842	2.925	0.156
Dynamic venous pressure (mmHg)	1.011	1.005	1.018	<0.001 *
*AGT*				
rs7079	1.781	0.623	5.093	0.282
rs11568056	1.003	0.649	1.548	0.990
rs6687360	1.205	0.824	1.761	0.336
rs4762	0.660	0.398	1.092	0.106
rs11568028	0.854	0.570	1.278	0.442
rs3789678	1.012	0.638	1.605	0.960
rs5051	0.922	0.609	1.398	0.703
*ACE*				
rs4295	1.303	0.833	2.036	0.246
rs4340	1.369	0.937	2.001	0.105
rs10853044	1.138	0.690	1.876	0.612
*AGTR1*				
rs409742	1.233	0.727	2.093	0.437
rs275653	1.393	0.881	2.203	0.157
rs10935724	0.902	0.596	1.366	0.626
rs1492099	1.437	0.846	2.441	0.180
rs385338	1.119	0.743	1.686	0.591
rs5182	1.285	0.852	1.937	0.231
rs5186	0.729	0.374	1.421	0.353
*AGTR2*				
rs1403543	1.014	0.699	1.472	0.940
rs11091046	0.970	0.652	1.444	0.882
rs12840631	1.105	0.726	1.684	0.641

*AGT*, Angiotensinogen; *ACE*, Angiotensinogen-converting enzyme; *AGTR1*, Angiotensin II receptor, type 1; *AGTR2*, Angiotensin II receptor, type 2; * *p* < 0.05.

**Table 4:** Univariate logistic regression model of factors associated with AVF malfunction in female HD patients.

Clinical or Genetic Factors	Odds Ratio	95% CI Lower	95% CI Upper	Significance
Age (year)	0.991	0.972	1.011	0.386
Right side *vs.* left side	0.766	0.406	1.445	0.411
Upper arm *vs.* forearm	2.690	1.371	5.279	0.004 *
Hypertension	0.606	0.348	1.055	0.077
Diabetes mellitus	0.664	0.342	1.287	0.225
Coronary artery disease	0.933	0.455	1.912	0.850
Peripheral artery disease	0.832	0.169	4.101	0.821
Cerebrovascular accident	1.500	0.494	4.552	0.474
Dynamic venous pressure (mmHg)	1.017	1.007	1.027	0.001 *
*AGT*				
rs7079	0.981	0.523	1.840	0.952
rs11568056	0.835	0.429	1.625	0.595
rs6687360	0.867	0.499	1.509	0.615
rs4762	0.510	0.234	1.110	0.090
rs11568028	0.766	0.413	1.423	0.399
rs3789678	1.310	0.662	2.590	0.438
rs5051	1.011	0.560	1.825	0.972
*ACE*				
rs4295	1.095	0.558	2.150	0.792
rs4340	1.401	0.805	2.439	0.233
rs10853044	1.520	0.723	3.197	0.269
*AGTR1*				
rs409742	0.856	0.366	2.003	0.720
rs275653	0.933	0.455	1.912	0.850
rs10935724	1.104	0.584	2.087	0.761
rs1492099	0.680	0.267	1.735	0.420
rs385338	1.204	0.669	2.167	0.535
rs5182	0.952	0.510	1.777	0.878
rs5186	0.483	0.179	1.304	0.151
*AGTR2*				
rs1403543	0.982	0.546	1.768	0.953
rs11091046	0.871	0.474	1.600	0.656
rs12840631	1.136	0.641	2.013	0.661

*AGT*, Angiotensinogen; *ACE*, Angiotensinogen-converting enzyme; *AGTR1*, Angiotensin II receptor, type 1; *AGTR2*, Angiotensin II receptor, type 2; * *p* < 0.05.

**Table 5:** Univariate logistic regression model of factors associated with AVF malfunction in male HD patients.

Clinical or Genetic Factors	Odds Ratio	95% CI Lower	95% CI Upper	Significance
Age (year)	1.014	0.997	1.031	0.115
Right side *vs.* left side	3.051	1.724	5.397	<0.0001 *
Upper arm *vs.* forearm	4.474	2.215	9.038	<0.0001 *
Hypertension	1.450	0.878	2.396	0.147
Diabetes mellitus	0.806	0.470	1.380	0.432
Coronary artery disease	1.374	0.771	2.447	0.281
Peripheral artery disease	1.603	0.564	4.555	0.376
Cerebrovascular accident	1.566	0.733	3.342	0.247
Dynamic venous pressure (mmHg)	1.008	1.000	1.017	0.061
*AGT*				
rs7079	0.984	0.547	1.768	0.956
rs11568056	1.185	0.668	2.107	0.563
rs6687360	1.615	0.951	2.744	0.076
rs4762	0.817	0.418	1.594	0.552
rs11568028	0.942	0.552	1.608	0.827
rs3789678	0.809	0.431	1.515	0.507
rs5051	0.850	0.472	1.530	0.588
*ACE*				
rs4295	1.459	0.799	2.663	0.219
rs4340	1.320	0.780	2.233	0.301
rs10853044	0.902	0.456	1.785	0.768
*AGTR1*				
rs409742	1.584	0.795	3.156	0.191
rs275653	1.900	1.035	3.489	0.038 *
rs10935724	0.785	0.452	1.361	0.388
rs1492099	2.287	1.160	4.509	0.017 *
rs385338	1.053	0.592	1.872	0.860
rs5182	1.623	0.938	2.808	0.084
rs5186	1.159	0.459	2.926	0.735
*AGTR2*				
rs1403543	1.099	0.661	1.826	0.717
rs11091046	1.133	0.654	1.964	0.656
rs12840631	1.145	0.598	2.191	0.683

*AGT*, Angiotensinogen; *ACE*, Angiotensinogen-converting enzyme; *AGTR1*, Angiotensin II receptor, type 1; *AGTR2*, Angiotensin II receptor, type 2; * *p* < 0.05.

**Table 6:** Multivariate logistic regression model of factors associated with AVF malfunction in male HD patients.

Clinical or Genetic Factors	Significance	Odds Ratio	95% CI Lower	95% CI Upper
Right side *vs.* left side	0.001 *	3.559	1.709	7.412
Upper arm *vs.* forearm	0.003 *	3.837	1.590	9.258
*AGTR1* rs1492099_CA+AA *vs.* CC	0.005 *	3.632	1.469	8.982
Age	0.543	1.007	0.985	1.029
Hypertension	0.313	0.702	0.353	1.395
Dynamic venous pressure	0.473	1.004	0.994	1.014
AGT rs6687360	0.065	1.885	0.962	3.692
AGTR1 rs409742	0.522	1.500	0.433	5.195
AGTR1 rs275653	0.913	0.917	0.195	4.307
AGTR1 rs5182	0.712	1.148	0.551	2.391

*AGT*, Angiotensinogen; *ACE*, Angiotensinogen-converting enzyme; *AGTR1*, Angiotensin II receptor, type 1; *AGTR2*, Angiotensin II receptor, type 2 * *p* < 0.05.

## Abbreviations

The following abbreviations are used in this manuscript:

ACE	Angiotensin converting enzyme
ACE-I	Angiotensin converting enzyme inhibitor
AGT	Angiotensinogen
AGTR-1/2	Angiotensin II receptor 1/2
ARB	Angiotensin receptor blocker
AVF	Arterio-venous fistula
CI	Confidence interval
ESRD	End stage renal disease
HTN	Hypertension
HD	Hemodialysis
HIF-1α	Hypoxia inducible factor-1α
HO-1	Heme oxygenase-1
MMPs	Matrix metalloproteinases
MTHFR	Methylene tetrahydrofolate reductase
NIH	Neo-intimal hyperplasia
OR	Odds ratio
PRMT1	Protein arginine methyltransferase 1
RAAS	Renin-angiotensin-aldosterone system
RRT	Renal replacement therapy
SNP	Single nucleotide polymorphism
TGF-β1	Transforming growth factor-β1
VEGF-A	Vascular endothelial growth factor-A
VEGFR-1/2	Vascular endothelial growth factor receptor-1/2
